# Comfort and Coordination among Interprofessional Care Providers Involved in Intubations in the Pediatric Intensive Care Unit

**DOI:** 10.1155/2023/4504934

**Published:** 2023-10-04

**Authors:** Chetna K. Pande, Kelsey Stayer, Thomas Rappold, Madeleine Alvin, Keri Koszela, Sapna R. Kudchadkar

**Affiliations:** ^1^Department of Pediatrics, Division of Critical Care Medicine, Baylor College of Medicine, Texas Children's Hospital, Houston, Texas, USA; ^2^Department of Anesthesiology and Critical Care Medicine, Children's Hospital of Philadelphia, University of Pennsylvania School of Medicine, Philadelphia, PA, USA; ^3^Department of Anesthesiology,Critical Care,and Pain Medicine, Boston Children's Hospital and Harvard Medical School, Boston, MA, USA; ^4^Department of Anesthesiology and Perioperative Medicine, Oregon Health and Science University, Portland, OR, USA; ^5^Department of Anesthesiology and Critical Care Medicine, Johns Hopkins University School of Medicine, Baltimore, Maryland, USA; ^6^Department of Pediatrics, Johns Hopkins University School of Medicine, Baltimore, Maryland, USA; ^7^Department of Physical Medicine & Rehabilitation, Johns Hopkins University School of Medicine, Baltimore, Maryland, USA

## Abstract

**Background:**

Successful execution of invasive procedures in acute care settings, including tracheal intubation, requires careful coordination of an interprofessional team. The stress inherent to the intensive care unit (ICU) environment may threaten the optimal communication and planning necessary for the safe execution of this complex procedure. The objective of this study is to characterize the perceptions of interprofessional team members surrounding tracheal intubations in the pediatric ICU (PICU).

**Methods:**

This is a single-center survey-based study of staff involved in the intubation of pediatric patients admitted to a tertiary level academic PICU. Physicians, nurses, and respiratory therapists (RT) involved in tracheal intubations were queried via standardized, discipline-specific electronic surveys regarding their involvement in procedural planning and overall awareness of and comfort with the intubation plan. Qualitative variables were assessed by both Likert scales and free-text comments that were grouped and analyzed thematically.

**Results:**

One hundred and eleven intubation encounters were included during the study time period, of which 93 (84%) had survey responses from at least 2 professional teams. Among those included in the analysis, the survey was completed 244 times by members of the PICU teams including 86 responses from physicians, 76 from nurses, and 82 from RTs. Survey response rates were >80% from each provider team. There were significant differences in interprofessional team comfort with nurses feeling less well informed and comfortable with the intubation plan and process compared to physicians and RTs (*p* < 0.001 for both). Qualitative themes including clear communication, adequate planning and preparation prior to procedure initiation, and clear definition of roles emerged among both affirmative and constructive comments.

**Conclusions:**

Exploration of provider perceptions and emergence of constructive themes expose opportunities for teamwork improvement strategies involving intubations in the PICU. The use of a preintubation checklist may improve organization and communication amongst team members, increase provider morale, decrease team stress levels, and, ultimately, may improve patient outcomes during this high stakes, coordinated event.

## 1. Introduction

Tracheal intubation is a common and life-saving intervention for children in the pediatric intensive care unit (PICU). Multicenter data indicate that in 20% of intubations, one or more tracheal intubation-associated adverse events (TIAEs) occur, which are associated with increased morbidity and mortality [1–3]. Successful execution of any procedure in the PICU requires coordination of care among several interprofessional team members including, but not limited to, physicians, registered nurses (RNs), advanced practice providers (APPs), respiratory therapists (RTs), and pharmacists. The acuity and stress inherent to the PICU environment may impede optimal communication and procedural planning which may, in turn, affect team performance and patient outcomes. This is particularly true in large academic centers, where trainees are the predominant bedside providers and are still learning the fundamentals of effective medical team management. In a study from an academic neonatal intensive care unit (NICU), higher team stress levels were associated with increased TIAEs during neonatal tracheal intubations [4].

Studies have shown that standardized protocols to ensure shared mental models improve communication and, ultimately, patient care, particularly in high-acuity settings. Two of the most well-recognized preprocedural bundles include the central venous catheter insertion checklist and the World Health Organization Surgical Safety Checklist which have both been implemented worldwide and have improved patient outcomes [5, 6]. In the PICU, explicit documentation of daily patient goals clarified plans among healthcare providers [7]. Furthermore, standardization of postoperative handover significantly reduced the need for patient-related hemodynamic or respiratory interventions and decreased antibiotic delays and the time to the first dose of analgesic medications [8]. In a recent multicenter series across 15 PICUs, the use of a tracheal intubation safety bundle significantly decreased TIAEs including both severe (such as cardiac arrest, emesis with aspiration, hypotension requiring intervention, laryngospasm, pneumothorax, or pneumomediastinum) and nonsevere (such as mainstem bronchial intubation, emesis without aspiration, epistaxis, medication error, and dental/lip trauma) [3].

Endotracheal intubation requires a complex choreography of the interprofessional team. Despite literature supporting the improved outcomes with greater communication surrounding high-risk procedures in medicine, to our knowledge, no studies exist describing interprofessional team perceptions regarding intubations in the PICU. By understanding the peri-intubation insights of different team members involved in the procedure, safety checklists may be tailored to maximize engagement for effective implementation and to improve the teamwork required for intubation success. Thus, the objective of this study was to characterize interprofessional perceptions surrounding the planning and execution of tracheal intubations in the PICU and determine if physicians, RNs, and RTs felt equally informed and comfortable regarding the intubation process.

## 2. Methods

### 2.1. Study Design

In this study, we aimed to describe the perceptions of interprofessional team members in the PICU surrounding intubations. The Institutional Review Board of the Johns Hopkins Hospital reviewed and acknowledged the study as quality improvement. All endotracheal intubation events occurring in our institution's PICU from May 2017 to May 2018 were included. At the time of data collection, no formal, standardized preintubation checklist was utilized.

### 2.2. Setting

Johns Hopkins Hospital's PICU is a 40-bed academic, tertiary care, combined medical-surgical unit with single-patient rooms. The unit is staffed by attending pediatric intensivists and pediatric anesthesiologists and houses a Pediatric Critical Care Medicine (PCCM) fellowship program. The RN-to-patient ratio is 1 : 1 to 1 : 2. There are multiple RTs on the unit at any given time with an average RT-to-patient ratio of 1 : 6. At any intubation, the minimum personnel present include the bedside RN, the assigned RT, and the laryngoscopist (usually a PCCM fellow in training supervised by an attending physician). Typically, the initial attempt is performed by a PCCM fellow, and if they are unsuccessful after 1-2 attempts, the attending provider or an advanced laryngoscopist, such as an anesthesiologist, attempts; this is based on the discretion of the attending provider.

### 2.3. Data Collection

Intubations were manually identified by the study investigators through twice daily surveillance of reports of intubated patients generated by the electronic health record (EHR). Once an intubation event was identified, EHR documentation was reviewed to determine the primary intubating provider, bedside RN, and primary RT involved in the procedure. Each of these team members was sent a questionnaire via secure institutional e-mail querying details of the intubation event (Supplemental Figures (available here)). Questionnaires were created by a multidisciplinary group of practitioners, including study team members and PICU staff. The goal was to collect both objective data about the intubation and subjective data regarding the responder's experience with the intubation. After multiple iterations, final versions were approved by leaders from all involved disciplines. Paper copies of the questionnaire were also available in the PICU for providers to fill out as an alternative. Questionnaires for intubating providers, RNs, and RTs included generalized questions and questions tailored to each discipline's unique role in the intubation process. These questionnaires were developed after conducting informal interviews with various staff in the PICU to gain a better understanding of the unique needs of different staff members, including attending physicians, PCCM fellows in training, representatives from nursing and RT leadership, as well as bedside RNs and RTs.

All team members were asked whether (1) the criteria to intubate were discussed prior to intubation; (2) they were aware of the entire intubation plan prior to the procedure; and (3) they were comfortable with the intubation process (specifically its planning and execution). Participants were then asked to rate on a Likert scale how well informed of and comfortable with the intubation plan they felt (1–10; 1, not well informed/not comfortable; 10, very well informed/very comfortable). The questionnaires also offered a section for participants to enter free-form comments regarding the intubation process and team communication.

### 2.4. Data Analysis

Data were analyzed using descriptive statistics. Categorical data were analyzed by the chi-square test, and continuous data (expressed as medians (interquartile range (IQR))) were analyzed using repeated-measure ANOVA. Study investigators manually verified the accuracy of staff-reported intubation details by cross-referencing data documented in the EHR. EHR documentation took precedence in cases of discord. Analyses were conducted in Stata 15, and a *p* value <0.05 was considered statistically significant. Free-text responses were reviewed by two investigators independently (CKP and KS), and responses were collated by theme.

## 3. Results

Of 111 intubation encounters during the study period, 93 (84%) included survey responses from at least two professional teams and were included in the analysis. The majority of events were oral intubations (96%) and occurred during the day shift (73%). Demographic data for patients are reported in Table 1. Overall, the cohort was 59% (*n* = 55) female, and the median age was 9 months of age (IQR: 3–84 months). The majority of patients were admitted to the PICU for respiratory disease and was intubated for respiratory failure. One hundred percent of intubations were ultimately successful, with the majority (67%) successful with one attempt. PCCM fellows in training were the primary laryngoscopist in 92% of intubations. TIAEs were reported in 28% (*n* = 26) of intubations.

**Figure 1:**
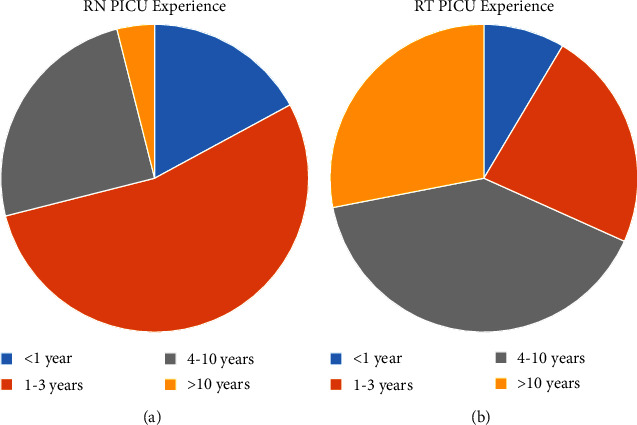
PICU RN and RT experience.

**Figure 2:**
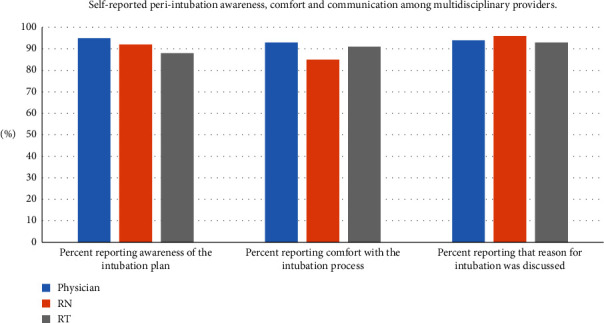
Awareness and comfort of team members with intubation process.

**Figure 3:**
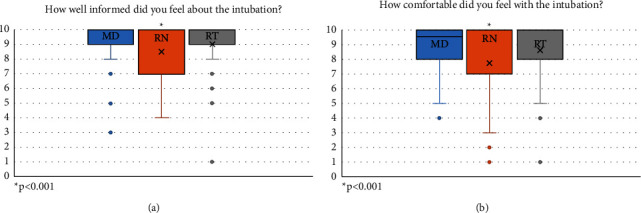
Comfort and awareness of team members with intubation process by a Likert scale.

Survey response rates were as follows: physicians 92% (*n* = 86), nurses 82% (*n* = 76), and RTs 88% (*n* = 82). Pediatric critical care fellows were the most common primary laryngoscopists and, therefore, constituted the majority of physician respondents. On average, RNs had less overall career experience (majority ≤3 years' experience) and experience in our PICU compared to RTs (majority ≥4 years' experience) (Figure 1). Physicians indicated awareness of the intubation plan 95% of the time, compared to nurses (92%) and RTs (88%; *p* = 0.29). Similarly, physicians (93%) and RTs (91%) indicated comfort with the intubation process more frequently than nurses, although not statistically significant (85%; *p* = 0.13). All groups reported an understanding of the reason for intubation in >90% of events (physicians: 94%, RNs: 96%; RTs: 93%; *p* = 0.89) (Figure 2). Regarding how comfortable they felt with the intubation process, physicians and RTs reported significantly higher scores compared with RNs with the physician median score 9.5 (IQR 8–10) compared to 8 (IQR 7–10) for RNs and 10 (IQR 8–10) for RTs (Figure 3; *p* < 0.001).

When asked how well informed they felt about the intubation plan, there were significant differences among the groups with the median score for physician 10 (IQR: 9-10), compared to 9 (IQR 7–10) for RNs and 10 (IQR 9-10) for RTs (Figure 3; *p* < 0.001).

### 3.1. Interprofessional Staff Free-Text Comments

In the free-form comments provided by physicians, RNs, and RTs, we identified several themes that characterize perceptions and experiences regarding the intubation process in our PICU, including (1) communication and teamwork, (2) planning and preparation, and (3) role identification. We will explore these themes in the following section with illustrative quotes that reflect both positive aspects of the intubation process and identify opportunities for improvement. Additional quotes from the questionnaires that further conceptualize the themes can be found in Table 2. Interestingly, physicians had the fewest comments related to the interprofessional nature of the intubation process. Rather, many physician comments addressed the medical aspects of the intubation, such as hemodynamic changes, descriptions of the airway, and medical reasons for multiple attempts.

#### 3.1.1. Theme 1: Communication and Teamwork

RNs and RTs, more often than physician providers, described poor communication amongst team members both prior to and during the intubation. When the procedure was perceived to go well, open communication and teamwork were specifically cited. Lack of communication reportedly increased confusion regarding the intubation plans during several events and may have contributed to medical errors during the intubation process. There were many suggestions for improving various logistical aspects of the intubation processes (such as medication administration and equipment availability), indicating that a shared mental model may not have always been present amongst team members.

Positive: “I felt the whole intubation went very smoothly and with great communication with the team.”—RT

Opportunity for Improvement: “Closed loop communication for meds to be given regarding rocuronium (roc) [could have been better]… Attending MD asked for roc, but intubating MD did not verify, so RN delayed administering it.”—RN

#### 3.1.2. Theme 2: Planning and Preparation

Each provider group frequently cited preprocedural planning and preparation as critical to procedure success or failure. When planning was well executed, team leaders were lauded for their detailed description of the plan, both when the decision to intubate was made and during the time-out immediately prior to intubation. Furthermore, when an intubation was well planned, the environment surrounding the procedure was often described as “controlled and calm” versus “hectic” when there was inadequate planning. Based on the comments, providers from each discipline felt more comfortable with the intubation process when the plan for intubation was described, including which medications were to be administered and in what order, what equipment would be used, and what backup plans would be implemented, if necessary. Providers also cited increased comfort when there was an opportunity for each team member to state their role and express any concerns prior to the intubation. If such discussion among interprofessional team members did not take place, it was often identified as an opportunity for improvement.

Positive: “Dr. [X] did a long time out explaining the procedure and complications to all staff involved. She ensured that we had all of the potential medications and equipment prior to the time out and during the time out. Everyone understood the risks associated with the patient and the plan if the intubation did not go as planned.”—RN

Opportunity for Improvement: “Clearer parameters for when the intubation would be done and what dosing for medication would be needed for the intubation [would have been helpful].”—RN

#### 3.1.3. Theme 3: Identification of Roles

Although one could argue that identification of roles during the procedure could be a product of communication or adequate preprocedural planning, this was often cited as a unique concern by RNs and RTs. Commentary detailed how, in emergency situations or when there were multiple physicians in the room, it was often difficult to identify the team leader, which lead to distress amongst interprofessional providers. Examples of role identification that were well received included identification of the primary laryngoscopist, the backup laryngoscopist should the first provider fail, the documenting RN, the RN administering mediations, the RT directly involved in the intubation, and providers available as extra help.

Positive: “Everyone's role was clear.”—RN

Opportunity for Improvement: “Unclear who was running code and managing qCPR”—RN

## 4. Discussion

In this study, we examined the perceptions of interprofessional team members involved in intubating critically ill children in an academic PICU. Although physicians, RNs, and RTs understood the reason for intubation, were aware of the plan, and felt comfortable with the intubation >80% of the time, we found a significant difference among the interprofessional staff comfort, with RNs feeling less well informed and comfortable with the intubation plan and process compared to physicians and RTs. Furthermore, qualitative comments suggest that overall comfort with the intubation was often contingent upon clear communication, plan discussion and preparation prior to procedure initiation, and clearly defined roles. Given the high risk of complications with procedures in critically ill patients and the variety of experience levels of interprofessional team members, checklists are increasingly being used to ensure adherence to safety practices, to promote a shared mental model, and to improve teamwork and communication [7, 9–15]. These findings suggest an opportunity to improve comfort amongst the interprofessional providers involved in the intubation of children in what is often a high-risk and high-stress environment.

We found that RNs felt less comfortable with the intubation process compared to physicians and RTs. It is not uncommon for physicians to develop a plan and relay this information to other team members [16–18]; however, it is surprising that there is a discrepancy between RN and RT providers, as both are critical to procedural success. Interestingly, the nursing cohort at the time of this study had less career experience in their field and in the PICU setting compared to the RTs, which may have contributed to the discrepancy seen in comfort level. Intuitively, one may expect that factors such as emergent procedures, procedures requiring multiple attempts, or procedures surrounding a change of shift may affect the perception of the procedure; however, these factors did not seem to negatively impact providers' perception of a procedure as long as there was effective communication among the team members and adequate preprocedural planning.

The need for clear communication and teamwork, adequate planning and preparation, and role identification are themes not unique to this study, as they have been frequently cited as key elements to success in high-acuity situations, both within and outside of medicine [9, 19–23]. It is well demonstrated that ineffective communication and teamwork directly compromise patient care, lead to staff distress, and are a contributory factor in the majority of hospital sentinel events [18, 22–26]. In fact, teamwork and leadership have been found to be so integral to cardiopulmonary resuscitation that they have been included in guidelines for advanced life support [10, 27]. In a pre-/postimplementation study assessing the utility of a PICU daily patient goal sheet, the use of the protocol improved both physicians' and nurses' perceived understanding of patient care goals as well as nurses' ability to accurately identify a patient's physician team [7]. Furthermore, a systematic review assessing the impact of a surgical safety checklist on the quality of teamwork and communication in the operating room found that safety checklists positively impact operating room teamwork and communication, which may, in turn, improve patient outcomes [11]. Moreover, as was seen in our study, communication and planning can contribute to a calm, controlled environment, which can, in turn, promote positive patient outcomes and success of procedures. In a large study of over 2,000 intubation events, high team stress levels were more frequently associated with adverse events, and low stress levels were highly correlated with high teamwork scores [4]. A potential solution to these identified opportunities for improvement may be the use of simulation in teamwork surrounding tracheal intubation [28].

With the success of safety checklists reducing morbidity and mortality in the operating room, much interest has been focused on improving periprocedure communication in similarly acute inpatient settings [6, 11]. While somewhat inconclusive, adult literature suggests intervention-targeted preprocedure protocols for endotracheal intubation can decrease life-threatening complications both in the emergency room and ICU [12, 13, 29]. Likewise, the use of standardized preintubation checklists, as well as electronic medication algorithms and order sets, improved patient safety in a large, academic NICU [14]. Finally, in a large multicenter time series study across PICUs in the United States, implementation of a tracheal intubation safety bundle, including airway safety preprocedural checklists, was associated with a decrease in TIAEs [3].

Efforts have been made to develop quality improvement bundles targeted to reduce TIAEs during pediatric intubation in PICUs [3], including the use of a standard preintubation checklist, though bundle compliance remained a significant barrier [30, 31]. It is important to note that it is not the mere presence of a checklist that may improve communication and reduce patient complications; rather, the checklist is a tool that, when utilized appropriately, can improve team communication [15, 32]. For example, although the use of a surgical safety checklist successfully decreased the rate of death and complications when implemented across eight worldwide cities, when that same checklist was implemented in hospitals across Canada, with reported 98% compliance, there was no reduction in mortality or complications [6, 33]. As such, when implementing a checklist, it is important to ensure buy-in from stakeholders and involve the interprofessional team members in its creation and execution.

Taken together, our study findings suggest that a structured, preintubation checklist may aid with organization and planning prior to intubation to improve provider comfort from and patient safety during the intubation process. During the COVID-19 pandemic, many ICUs mandated a minimum amount on personnel in the room with the door closed, which only highlights the need for clear communication and preparation prior to any procedures. By providing dedicated time during which team members may offer critical patient-related information, flag knowledge gaps, and promote coordination and shared decision-making, interprofessional team members may deliver better care. Though, we acknowledge and recognize that in emergent situations, going through a checklist may not be feasible for the safety of the patient. As with many quality improvement efforts, effective implementation depends on broad stakeholder engagement from interprofessional team members.

There are several limitations to this study. The results are based on the experience of a single-center tertiary care PICU, which may limit the generalizability of our findings. Although we followed a process to obtain accurate data, because our data are self-reported, there is a possibility of recall bias. Furthermore, given that there are a finite number of personnel to be involved in multiple intubations, this may be a confounding factor that we did not adjust for. Finally, it is unique that we perform nasal intubations in our PICU; although it is a more common event in our PICU compared to other PICUs, it is still rare and may have contributed at least in part to the abundant commentary regarding opportunities for improvement surrounding equipment.

## 5. Conclusion

In this study assessing the perceptions of interprofessional team members regarding the intubation process at a large academic PICU, RNs, RTs, and MDs reported awareness of and comfort with the intubation process >80% of the time. However, RNs reported significantly less awareness and comfort compared to RTs and MDs. Upon assessment of free-form comments regarding the intubation process, several themes emerged including communication and teamwork, preprocedural planning and preparation, and role identification. A structured preintubation checklist may help to improve communication, procedural planning, and role identification, creating a shared mental model, improving comfort and stress levels amongst interprofessional team members, and ultimately ensuring patient safety.

## Figures and Tables

**Table 1:** Patient characteristics.

Characteristics, *n* (%) or median (IQR)	Study population (*n* = 93)
Age, months	9 (3, 84)
Female sex	55 (59)
Admission category	
Respiratory	25 (27)
Shock/sepsis	8 (9)
Cardiac (medical)	16 (17)
Trauma	1 (1)
Neurologic	15 (16)
Surgical	19 (20)
Others	6 (6)
Missing	3 (3)
Intubation indication	
Oxygenation failure	31 (33)
Ventilation failure	16 (17)
Hemodynamic instability	10 (11)
Neuromuscular weakness	4 (4)
Upper airway obstruction	4 (4)
Pulmonary toilet	4 (4)
Impaired airway reflex	5 (5)
Elective	14 (15)
Others	1 (1)
Unplanned extubation	2 (2)
Missing	2 (2)
Time of intubation	
Day shift	68 (73)
Night shift	25 (27)
Difficult airway	20 (22)
Urgency of intubation	
Emergent (intubation without delay)	42 (45)
Urgent (intubation within 1 hour)	29 (31)
Nonurgent (intubation in >1 hour)	20 (22)
Unknown	2 (2)
Time-out performed	73 (78)
Oral intubation	89 (96)

**Table 2:** Selected quotes from interprofessional team members.

	Positive	Opportunity for improvement
Communication	“The fellow made it clear that we needed to intubate. Both fellow and attending agreed on drugs needed and verbally confirmed them with the bedside and charge nurses”—RN “I felt very comfortable and felt this intubation went very well…The whole team communicated well!”—RT “It was surprisingly well-coordinated for an unplanned reintubation, good communication”—MD	“Communication between [teams]…could have been better.”—RN “RT was never informed about intubating the patient or the severity of the patients condition until they made an urgent request for the vent and end tidal to be in the room”—RT “Also forgot to notify RT in time so a delay getting end tidal and vent set up when we were all ready to intubate”—MD

Planning and preparation	“The intubation was planned and went smoothly. It was an intubation we had been anticipating the need for several hours so we were very well prepared.”—RN “The intubation went really smoothly, we had all our equipment at the bedside we needed…”—RN “I think the intubation went very smoothly every thing was discussed before hand and during a time out”—RT “MD did an excellent job communicating his concerns and plan and back-up plan prior to the patient being induced for intubation.”—RT “Intubation was well executed with time out, check that all medications and equipment were in the room and closed loop communication”—MD	“Planning should have involved the bedside RN”—RN “MD asked for equipment which wasn't discussed”—RT “The patient's airway was larger than expected and we needed to upsize our blade and I went to the door and asked someone to bring us said blade, not knowing that the MD had placed it on the back of the boom. If I had been present before the actual start of the procedure I believe I would have known this”—RT “More meds drawn up in advance”—MD

Role identification	“All personnel were present”—RN	“It could have been more organized. We were receiving orders from 2 different attendings.”—RN “There were many doctors at the bedside during this intubation…it was unclear who was to be giving orders for which medication to be given.”—RN

## Data Availability

The data used to perform this study are not publicly available to protect patient and staff privacy.

## Supplementary Materials

Supplementary Materials Supplementary Figures: preintervention data collection forms sent to physicians, RNs, and RTs after every intubation event.
