# Supplementary material for: Comfort and Coordination among Interprofessional Care Providers Involved in Intubations in the Pediatric Intensive Care Unit
Source: Crit Care Res Pract. 2023 Oct 4;2023:4504934. doi: 10.1155/2023/4504934 (PMC10567513; doi:10.1155/2023/4504934)
Supplement: Supplementary Materials — Supplementary Figures: preintervention data collection forms sent to physicians, RNs, and RTs after every intubation event. [file 4504934.f1.zip › Tracheal Intubation Study Provider Form.pdf]

Reducing Tracheal Intubation Associated Events in the PICU
Quality Improvement Project

Physician

Pre-Intervention Data Collection Form

Intubation Planning

1) **1° reason for PICU admission**:
- ☐ Upper respiratory disease
- ☐ Lower respiratory disease
- ☐ Cardiac (medical)
- ☐ Cardiac (surgical)
- ☐ Shock/sepsis
- ☐ Neurological
- ☐ Trauma
- ☐ Other: ___________________________

2) **1° indication for intubation**:
- ☐ Oxygenation failure
- ☐ Ventilation failure
- ☐ Therapeutic hyperventilation
- ☐ Neuromuscular weakness
- ☐ Impaired airway reflex
- ☐ Upper airway obstruction
- ☐ Hemodynamic instability
- ☐ Elective procedure
- ☐ Pulmonary toilet
- ☐ Other: ___________________________

3) **Urgency of intubation**:
- ☐ Intubation without delay
- ☐ Intubation within 1 hour
- ☐ Intubation >1 hour

4) **Features associated with difficult airway (check all that apply)?**
- ☐ None
- ☐ Midface hypoplasia
- ☐ Limited mouth opening
- ☐ Small thyromental space
- ☐ Limited neck extension
- ☐ Other: ___________________________
- ☐ Upper airway obstruction

5) **Intubating provider(s):**

Provider #1	***Provider #2*** *(if applicable)*	***Provider #3*** *(if applicable)*
Level of training:	*Level of training*:	*Level of training*:
☐ Resident ☐ Fellow	☐ Resident ☐ Fellow	☐ Resident ☐ Fellow
☐ Attending ☐ NP	☐ Attending ☐ NP	☐ Attending ☐ NP
☐ Med student	☐ Med student	☐ Med student
Discipline:	*Discipline*:	*Discipline*:
☐ Peds ☐ Anes	☐ Peds ☐ Anes	☐ Peds ☐ Anes
☐ Ped/Anes ☐ ED	☐ Ped/Anes ☐ ED	☐ Ped/Anes ☐ ED
Prior successful intubations:	*Prior successful intubations*:	*Prior successful intubations*:
☐ None ☐ 1-5	☐ None ☐ 1-5	☐ None ☐ 1-5
☐ 6-10 ☐ 11-39	☐ 6-10 ☐ 11-39	☐ 6-10 ☐ 11-39
☐ 40+	☐ 40+	☐ 40+

Intubation Event

1) **Time of intubation:**
- ☐ Date: __________________________
- ☐ Time: ____________________________

2) **Route of intubation**:
- ☐ Oral
- ☐ Nasal

3) **How many times was intubation attempted* by each provider?**
*Attempt: A single advanced airway maneuver (e.g., beginning with insertion of laryngoscope into patient's mouth and ending when scope is removed).

Provider #1: ____________________ | ***Provider #2*** *(if applicable):* ___________________ | ***Provider #3*** *(if applicable):* _________________

4) **Which provider successfully placed the endotracheal tube?**
- ☐ Provider #1
- ☐ Provider #2
- ☐ Provider #3

5) **How was intubation confirmed (check all that apply)?**
- ☐ Direct visualization of ETT passing through vocal cords
- ☐ Chest rise
- ☐ Auscultation
- ☐ ETCO2 detection
- ☐ CXR

OVER

6) **a. Were there any desaturations*?** ☐ Yes ☐ No

*Desaturation: SpO2< 90% (in nonmixing patients) or drop in SpO2 >10% below baseline SpO2 (in mixing patients).

b. If so, how many? ______________________________

7) **Was there bradycardia*?** ☐ Yes ☐ No

*Bradycardia: Drop in heart rate >20% below baseline heart rate.

8) **Was a timeout performed with all team members present and attentive?** ☐ Yes ☐ No

9) **a. Were there any immediate complications that occurred with intubation?** ☐ Yes ☐ No

b. If yes, please check all that apply:

Severe Complications

- ☐ Cardiac arrest requiring >1 minute of chest compressions*
- ☐ Esophageal intubation with delayed recognition leading to deterioration in patient condition*
- ☐ Hypotension (drop of MAP >20% below goal) requiring Intervention*
- ☐ Emesis with aspiration
- ☐ Laryngospasm*
- ☐ Malignant hypertension*
- ☐ Pneumothorax or pneumomediastinum
- ☐ Dental or gum trauma
- ☐ Direct airway injury

Non-Severe Complications

- ☐ Mainstem bronchial intubation
- ☐ Esophageal intubation with immediate recognition
- ☐ Hypertension requiring intervention*
- ☐ Emesis without aspiration
- ☐ Epistaxis
- ☐ Lip trauma
- ☐ Medication error
- ☐ Bradycardia*
- ☐ Other dysrhythmia*
- ☐ Pain or agitation requiring additional medications and delaying intubation

*If any equipment or medications were used to correct the above complications, please indicate this on questions 4 and 5 below.

Process Improvement

1) **a. Were you aware of the entire intubation plan before the start of the procedure?** ☐ Yes ☐ No

b. On a scale of 1-10, did you feel well-informed about the intubation plan? ______________________

1 = Not well informed, 5 = Fairly informed with room for improvement, 10 = Very well informed, comfortable with plan

2) **a. Do you think the intubation process could have gone more smoothly?** ☐ Yes ☐ No

b. On a scale of 1-10, how smoothly do you think the intubation process went? ______________________

*Please consider the pre/post-intubation periods as well as the time during which intubation was occurring.

1 = Not smoothly at all, 5 = Fairly smoothly with room for improvement, 10 = Very smoothly, no improvements necessary

3) **Were the criteria to intubate discussed prior to intubation?** ☐ Yes ☐ No

4) **Was any additional *equipment* required during the intubation that was not present at the bedside prior to starting intubation? If so, what?** ☐ Yes ______________________________ ☐ No

5) **Were any additional *medications* required during the intubation that were not present at the bedside prior to starting intubation? If so, which?** ☐ Yes ______________________________ ☐ No

6) **Other comments about the intubation process:** ______________________________

____

Please place patient label here (or provide MRN).
