# Supplementary material for: Comfort and Coordination among Interprofessional Care Providers Involved in Intubations in the Pediatric Intensive Care Unit
Source: Crit Care Res Pract. 2023 Oct 4;2023:4504934. doi: 10.1155/2023/4504934 (PMC10567513; doi:10.1155/2023/4504934)
Supplement: Supplementary Materials — Supplementary Figures: preintervention data collection forms sent to physicians, RNs, and RTs after every intubation event. [file 4504934.f1.zip › Tracheal Intubation Study RN Form.pdf]

Reducing Tracheal Intubation Associated Events in the PICU
Quality Improvement Project

Bedside Nurse

Pre-Intervention Data Collection Form

Nursing Experience

1) **Nursing experience:**	☐ <1 year	☐ 1-3 years	☐ 4-10 years	☐ >10 years
2) **PICU experience:**	☐ <1 year	☐ 1-3 years	☐ 4-10 years	☐ >10 years
3) **JHH PICU experience:**	☐ <1 year	☐ 1-3 years	☐ 4-10 years	☐ >10 years

Patient Information

1) **Is this patient a known difficult airway**?	☐ Yes	☐ No	☐ Unknown
2) **Is this patient a documented difficult airway in Epic?**	☐ Yes	☐ No	☐ N/A

Intubation Event

1) **What medications were administered during the intubation process?**

☐ Atropine	☐ Dexmedetomidine	☐ Etomidate	☐ Fentanyl	☐ Glycopyrrolate	☐ Ketamine
☐ Lidocaine	☐ Midazolam	☐ Propofol	☐ Rocuronium	☐ Succinylcholine	☐ Vecuronium

☐ Other(s): ____

Process Improvement

1) **a. Were you aware of the entire intubation plan before the start of the procedure?** ☐ Yes ☐ No

b. On a scale of 1-10, did you feel well-informed about the intubation plan? ______________________
1 = Not well informed, 5 = Fairly informed with room for improvement, 10 = Very well informed, comfortable with plan

2) **a. Do you think the intubation process could have gone more smoothly?** ☐ Yes ☐ No

b. On a scale of 1-10, how smoothly do you think the intubation process went? ______________________
*Please consider the pre/post-intubation periods as well as the time during which intubation was occurring.
1 = Not smoothly at all, 5 = Fairly smoothly with room for improvement, 10 = Very smoothly, no improvements necessary

3) **Were the criteria to intubate discussed prior to intubation?** ☐ Yes ☐ No

4) **a. Was any additional *equipment* required during the intubation that was not present at the bedside prior to intubation?** ☐ Yes ☐ No

b. If so, what equipment? ____

5) **a. Were any additional *medications* required during the intubation that were not present at the bedside prior to intubation?** ☐ Yes ☐ No

b. If so, which medicines? ____

6) **Other comments about the intubation process:** ____

May also use back of page (just indicate with arrow).

Please place patient label here (or provide MRN).
