# Supplementary material for: Comfort and Coordination among Interprofessional Care Providers Involved in Intubations in the Pediatric Intensive Care Unit
Source: Crit Care Res Pract. 2023 Oct 4;2023:4504934. doi: 10.1155/2023/4504934 (PMC10567513; doi:10.1155/2023/4504934)
Supplement: Supplementary Materials — Supplementary Figures: preintervention data collection forms sent to physicians, RNs, and RTs after every intubation event. [file 4504934.f1.zip › Tracheal Intubation Study RT Form.pdf]

Reducing Tracheal Intubation Associated Events in the PICU
Quality Improvement Project

Respiratory Therapist

Pre-Intervention Data Collection Form

RT Experience

1) **RT experience:** ☐ <1 year ☐ 1-3 years ☐ 4-10 years ☐ >10 years
2) **PICU experience:** ☐ <1 year ☐ 1-3 years ☐ 4-10 years ☐ >10 years
3) **JHH PICU experience:** ☐ <1 year ☐ 1-3 years ☐ 4-10 years ☐ >10 years

Intubation Event

1) **a. Was optimal positioning of the patient discussed prior to intubation?** ☐ Yes ☐ No

   b. Was optimal positioning of the patient achieved during intubation? ☐ Yes ☐ No

   c. Was it necessary to reposition the patient after the start of intubation? ☐ Yes ☐ No

Process Improvement

1) **a. Was all of the respiratory equipment checked prior to intubation?** ☐ Yes ☐ No

   b. Was all necessary respiratory equipment at the bedside prior to intubation? ☐ Yes ☐ No

2) **a. Was the patient pre-oxygenated using a tightly sealed face mask?** ☐ Yes ☐ No

   b. If so, for approximately how long? ______________________

3) **a. Were you aware of the entire intubation plan before the start of the procedure?** ☐ Yes ☐ No

   b. On a scale of 1-10, did you feel well-informed about the intubation plan? ____________________________
   1 = Not well informed, 5 = Fairly informed with room for improvement, 10 = Very well informed, comfortable with plan

4) **a. Do you think the intubation process could have gone more smoothly?** ☐ Yes ☐ No

   b. On a scale of 1-10, how smoothly do you think the intubation process went? ___________________________
   *Please consider the pre/post-intubation periods as well as the time during which intubation was occurring.
   1 = Not smoothly at all, 5 = Fairly smoothly with room for improvement, 10 = Very smoothly, no improvements necessary

5) **Were the criteria to intubate discussed prior to intubation?** ☐ Yes ☐ No

6) **Other comments about the intubation process:** ____

   ____

Please place patient label here (or provide MRN).
